# 3D superconducting hollow nanowires with tailored diameters grown by focused He^+^ beam direct writing

**DOI:** 10.3762/bjnano.11.104

**Published:** 2020-08-11

**Authors:** Rosa Córdoba, Alfonso Ibarra, Dominique Mailly, Isabel Guillamón, Hermann Suderow, José María De Teresa

**Affiliations:** 1 Instituto de Ciencia Molecular, Universitat de València, Catedrático José Beltrán 2, 46980 Paterna, Spain; 2 Laboratorio de Microscopías Avanzadas (LMA)-Instituto de Nanociencia de Aragón (INA), Universidad de Zaragoza, E-50018 Zaragoza, Spain Departamento de Física de la Materia Condensada, Universidad de Zaragoza, E-50009 Zaragoza, Spain; 3 Centre de Nanosciences et de Nanotechnologies, CNRS, Univ Paris Sud, Université Paris Saclay, 91120 Palaiseau, France; 4 Laboratorio de Bajas Temperaturas y Altos Campos Magnéticos, Departamento de Física de la Materia Condensada, Instituto de Ciencia de Materiales Nicolás Cabrera, Condensed Matter Physics Center (IFIMAC), Universidad Autónoma de Madrid, 28049, Madrid, Spain; 5 Instituto de Nanociencia y de Materiales de Aragón (INMA), Universidad de Zaragoza-CSIC, E-50009 Zaragoza, Spain; 6 Departamento de Física de la Materia Condensada, Universidad de Zaragoza, E-50009 Zaragoza, Spain

**Keywords:** electron tomography, focused ion beam induced deposition (FIBID), helium ion microscope, magneto-transport measurements, nano-superconductors, tungsten carbide (WC)

## Abstract

Currently, the patterning of innovative three-dimensional (3D) nano-objects is required for the development of future advanced electronic components. Helium ion microscopy in combination with a precursor gas can be used for direct writing of three-dimensional nanostructures with a precise control of their geometry, and a significantly higher aspect ratio than other additive manufacturing technologies. We report here on the deposition of 3D hollow tungsten carbide nanowires with tailored diameters by tuning two key growth parameters, namely current and dose of the ion beam. Our results show the control of geometry in 3D hollow nanowires, with outer and inner diameters ranging from 36 to 142 nm and from 5 to 28 nm, respectively; and lengths from 0.5 to 8.9 µm. Transmission electron microscopy experiments indicate that the nanowires have a microstructure of large grains with a crystalline structure compatible with the face-centered cubic WC_1−_*_x_* phase. In addition, 3D electron tomographic reconstructions show that the hollow center of the nanowires is present along the whole nanowire length. Moreover, these nanowires become superconducting at 6.8 K and show high values of critical magnetic field and critical current density. Consequently, these 3D nano-objects could be implemented as components in the next generation of electronics, such as nano-antennas and sensors, based on 3D superconducting architectures.

## Introduction

Superconductors are dissipationless carriers of electric current and provide macroscopic, and thus robust, quantum coherence. This allows for a wide range of applications, particularly at the nanometer-scale, where they can be easily integrated in circuits and used as ultrasensitive sensors of magnetic fields, temperature and as key elements for quantum computation. The behavior of nanosized superconductors as one-dimensional quantum oscillators [1], Josephson junction arrays [2], electronic transport devices [3–7], very small-scale devices [8–9], micrometer-scale coolers [10], or thermal and spin sensors [11–12] has been studied in detail.

Nowadays, research on manufacturing highly energy-efficient three-dimensional (3D) structures [13] is critical for the development of future electronics. However, when approaching the nanometer-scale, the number of works on real 3D nano-superconductors [14–19] decreases dramatically, mostly due to the complex fabrication and characterization. A technique successfully utilized for fabricating 3D nano-objects is direct writing by a focused beam of positively charged particles, the so-called focused-ion-beam induced deposition (FIBID) [20]. A very promising development of FIBID is based on Ga^+^ ions. Functional 3D nanomaterials have been grown by Ga^+^ FIBID in the last decade [21–26]. In particular, Ga^+^ FIBID in combination with W(CO)_6_ as precursor material yielded 3D superconducting W-based wires with a critical temperature (*T*_c_) below 5 K and a critical magnetic field (µ_0_*H*_c2_(0)) up to 9.5 T [14–16]. Alternatively, in combination with Nb(NMe_2_)_3_(N-*t*-Bu), Ga^+^ FIBID yielded NbC wires with a broadened *T*_c_ range from 4 to 11 K [18]. One significant limitation is that 3D elements below 100 nm in diameter cannot be obtained with Ga^+^ FIBID, mainly due to the relatively large Ga^+^ beam diameter (approx. 5 nm) and a high proximity effect generated by Ga^+^ ion scattering.

Regarding a higher spatial resolution, the helium ion microscope (HIM) [27], based on a gas field-ionization source, has emerged as a tool for direct writing of complex 3D nano-objects taking advantage of its small beam diameter (approx. 0.3 nm) and low proximity effect [28]. When precursor molecules from the gas phase are adsorbed on a substrate surface, He^+^ FIB dissociates them into non-volatile and volatile products. The non-volatile products attach to the surface, resulting in a deposit, whereas the volatile products ones are pumped out of the process chamber. Normally, the final deposit is a mixture of carbon, metallic elements and oxygen. As clearly described using analytical modelling [29] and Monte-Carlo simulations [30], the vertical growth of 3D nano-objects by He^+^ FIBID is mainly caused by secondary electrons of the first order produced from the primary ion beam, whereas the lateral growth is induced by secondary electrons of the second order generated from scattered ions. Thus, the direct contribution of the primary He^+^ ion beam and the scattered He^+^ ions is almost negligible for the growth of these 3D nano-objects. Nevertheless, it is worth mentioning that its resolution, volume per dose and throughput are very sensitive to the selected growth conditions such as ion beam energy, ion beam current, precursor flux, surface interactions with the beam, and precursor molecules [29–30]. Hence, the He^+^ FIBID technique is highly recommended for direct writing of 3D nano-objects with high resolution and aspect ratio [17,19,31–35]. A successful example of tailored 3D nano-objects grown by He^+^ FIBID has been reported by Kohama and co-workers [35]. The authors deposited W-based pillars with diameters down to approx. 40 nm and aspect ratios of approx. 50. The microstructure of the grown material consisted of fcc WC_1−_*_x_* and W_2_(C,O) grains. Moreover, when the He^+^ beam was well focused the authors observed columnar voids created at the center of the pillars with a diameter ranging from 1 to 15 nm, showing the path to build complex 3D nano-objects beyond standard nanowires (NWs). Recent breakthroughs in the growth of 3D WC superconducting nano-objects with extremely large aspect ratios using He^+^ FIBID have been reported by some of the authors, such as hollow NW-like nanotubes as small as 32 nm in diameter [17] and nanohelices with controllable geometries, including the smallest and most densely packed nanohelix to date with a diameter of 100 nm [19].

In this work, we present the direct writing of 3D WC crystalline superconducting hollow NWs with tailored diameters grown using a HIM. The hollow NW geometry is successfully controlled by tuning the ion beam current and dose from 0.65 to 7 pA and from 0.1 to 0.4 nC, respectively, resulting in NWs with outer diameters from 36 to 142 nm and with inner diameters from 5 to 28 nm, and total length from 0.5 to 8.9 µm (aspect ratio ≈ 196). These values are significantly better than those reported in previous works [17,35]. The NWs microstructure consists of large grains of fcc WC_1−_*_x_*, in good agreement with [17,35]. In addition, the NWs are hollow along the whole NW length, which could make them nonconventional nanopipettes, as demonstrated in 3D reconstructions of electron tomography experiments. Finally, these 3D hollow NWs exhibit superconductivity below 6.8 K (*T*_c_), as well as high upper critical magnetic fields µ_0_*H*_c2_ ≈ 14.7 T, and large critical current densities *J*_c_ ≈ 0.15 MA/cm^2^.

## Results and Discussion

### Growth of 3D hollow nanowires by He^+^ FIBID

We use a HIM in combination with a W(CO)_6_ precursor to grow individual, out-of-plane WC NWs in a single step, controlling inner and outer diameter and total length. The precursor gas is delivered to the process chamber and adsorbed onto the substrate surface, while the He^+^ FIB spot remains fixed during the deposition favoring continuous vertical growth along [17].

#### Dimensional control for nanowires

**Figure 1:**
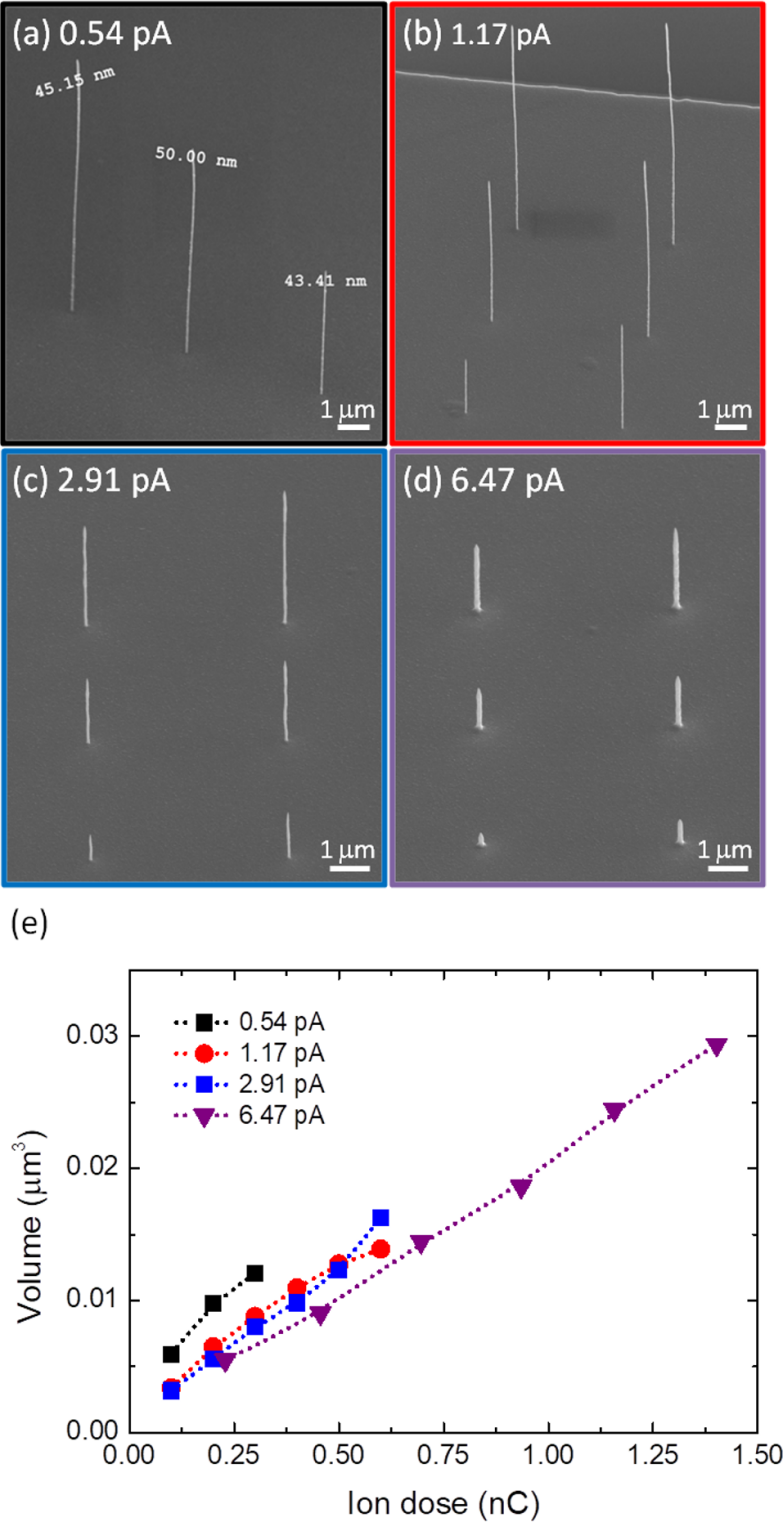
(a–d) SEM images of hollow NWs grown by He^+^ FIBID; (a) ion beam current: 0.54 pA, dose: 0.1–0.3 nC from right to left; (b) ion beam current: 1.17 pA, dose: 0.1–0.6 nC from left to right and upwards; (c) ion beam current: 2.91 pA, dose: 0.1–0.6 nC from left to right and upwards; (d) ion beam current: 6.47 pA, dose: 0.229, 0.456, 0.696, 0.935, 1.158, and 1.402 nC from left to right and upwards. (e) NW volume in (a) as a function of the ion beam dose.

We investigated the dimensional control for these NWs by optimizing in the deposition the following parameters: the ion beam current and ion dose. SEM images of typical NWs grown with ion beam currents ranging from 0.54 to 6.47 pA and doses from 0.1 to 1.4 nC are depicted in Figure 1a–d.

**Figure 2:**
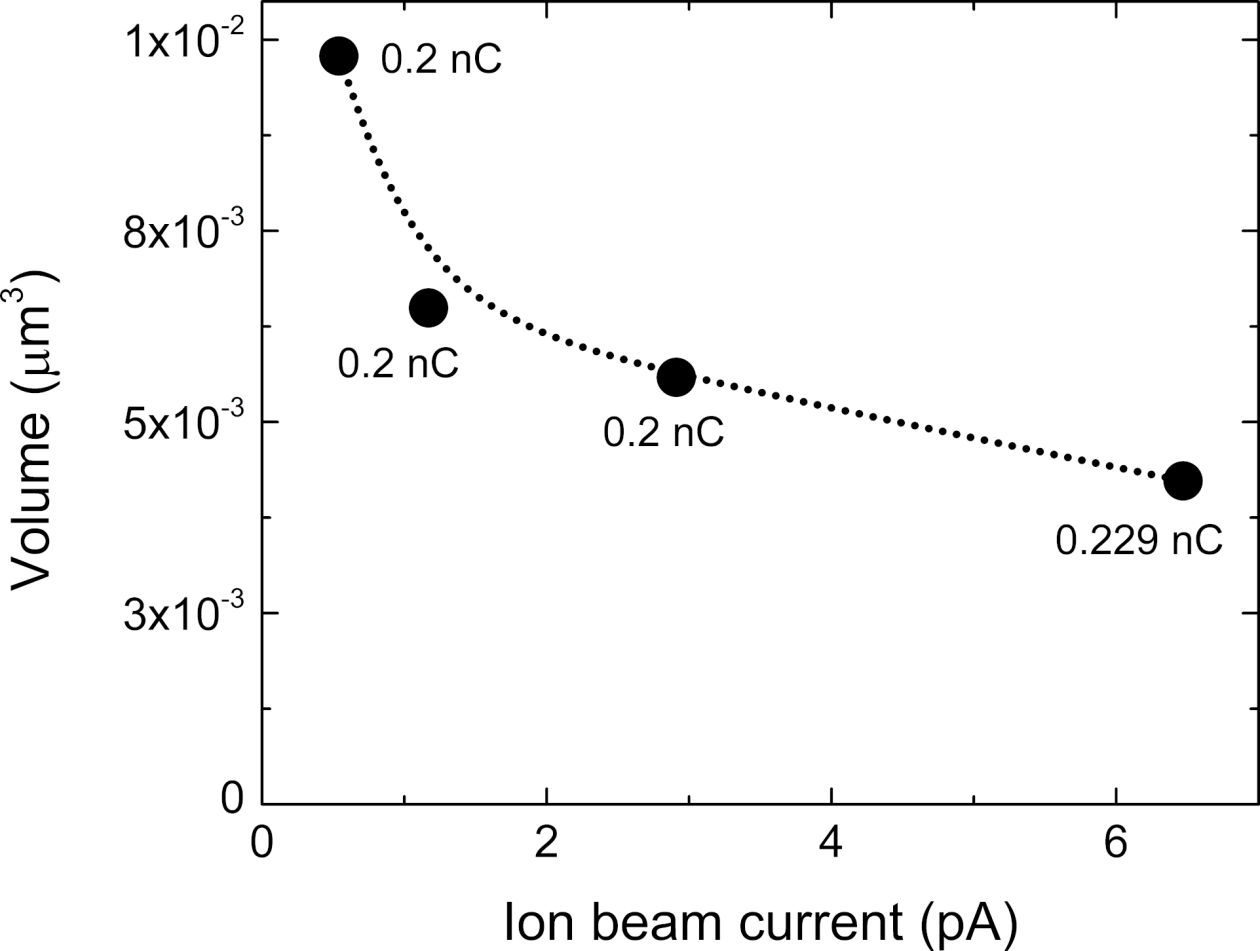
NW volume for a specific dose, as a function of the ion beam current.

Varying these parameters enables us to fabricate 3D NWs with diameters ranging from 45 to 125 nm, lengths ranging from 0.5 to 8.9 µm, and with aspect ratios up to 198. Further details regarding the growth conditions are described in the Experimental section. We found a linear dependence of the NW volume (determined as π × (outer diameter/2 – inner diameter/2)^2^ × height) as a function of the ion dose for the mentioned ion beam currents (Figure 1e). Moreover, we noted that the NW volume rapidly decreases as a function of the ion beam current for the same dose (Figure 2). When using high currents several effects can play a role in this dependence such as precursor depletion, local heating, which decreases the precursor molecule sticking coefficient, and low precursor diffusion from the substrate to the top of the pillar [36–37]. This shows the need for future systematic experiments varying the dwell time in pulsed growth or varying the flux of precursor gas.

### (High-resolution) scanning transmission electron microscopy

#### Dependence of NW inner diameter on the ion beam current

**Figure 3:**
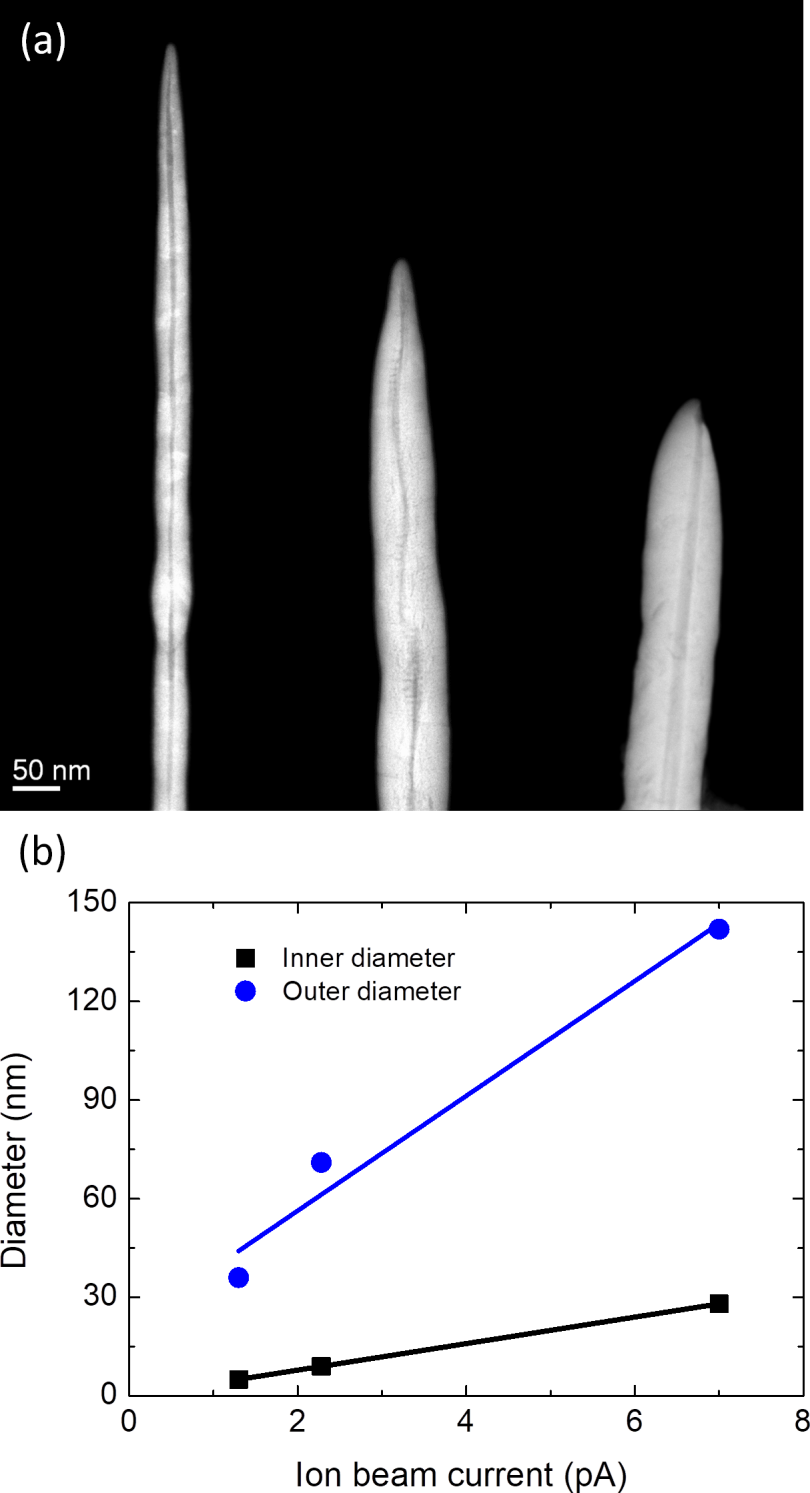
(a) STEM images of hollow NWs grown at 1.3, 2.3, and 7 pA, from left to right. (b) NW diameter in (a) as a function of the ion beam current.

To investigate the dependence of the NW inner diameter on the ion beam current, scanning transmission electron microscopy (STEM) experiments were performed. We found that inner diameter of the hollow NWs changes from 5 to 28 nm, whereas the outer diameter changes from 36 to 143 nm upon increasing the ion beam current from 1.3 to 7 pA. STEM images of these hollow NWs are shown in Figure 3a. The observed non-uniform shape of the cavity in the central nanowire could be explained by several reasons, such as He^+^ FIB instability or irregular substrate surface. We find a linear dependence of the inner diameter on the ion beam current (Figure 3b), which indicates that the ion beam forms the cavity due to a milling effect. Thus via tuning the ion beam current and dose we have full control to tailor the diameters of the hollow 3D NWs. The specific deposition parameters and NW diameters are listed in Table 1.

**Table 1:** Growth parameters and diameters of hollow WC NWs.

hollow nanowire type	1	2	3

ion beam current (pA)	1.3	2.3	7.0
outer diameter (nm)	36	71	142
inner diameter (nm)	5	9	28

#### Electron tomography

**Figure 4:**
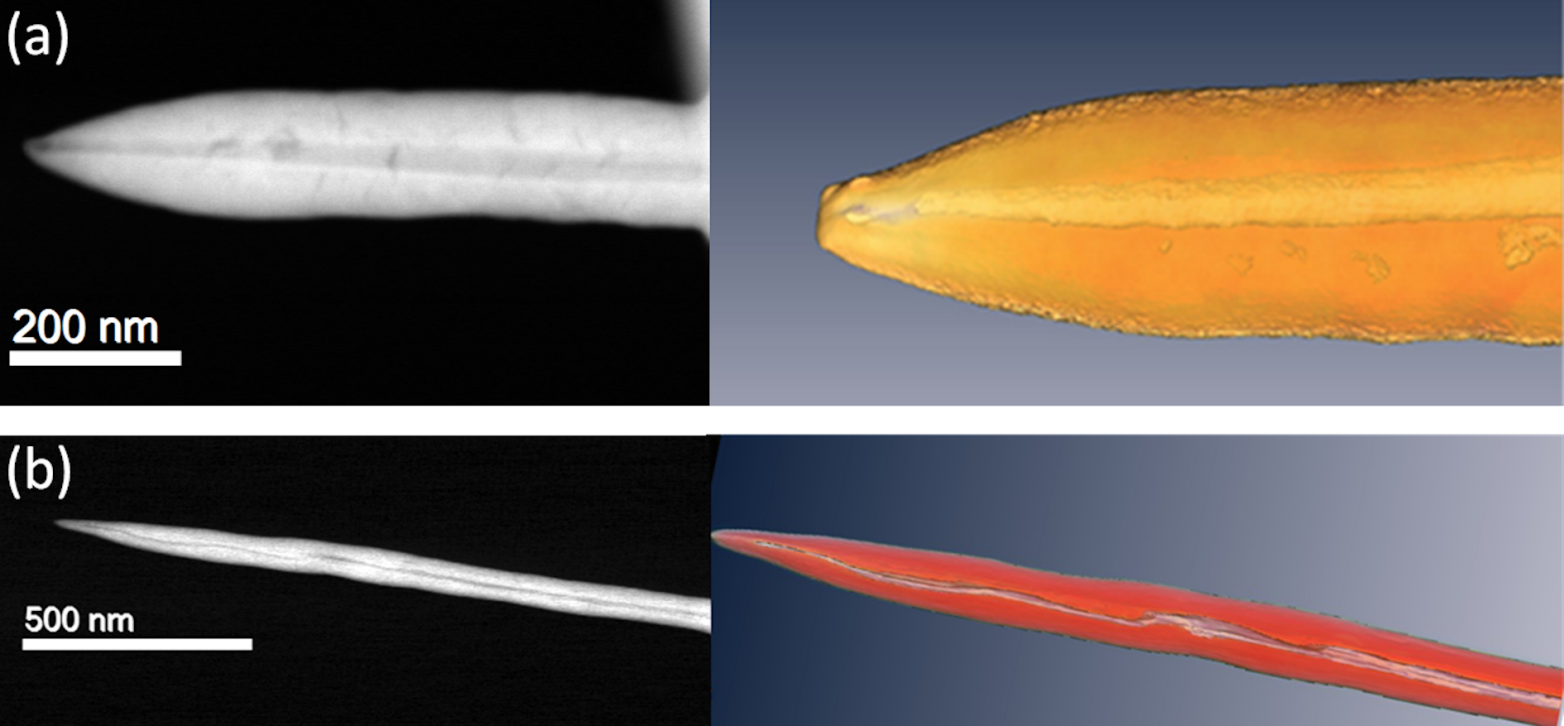
(a) Tomography of a hollow WC NW grown at 7 pA, with an outer diameter of 142 nm and an inner diameter of 28 nm; left panel: STEM image, right panel: snapshot of the 3D tomographic reconstruction. (b) Tomography of a hollow WC NW grown at 2 pA, with an outer diameter of 77 nm and inner diameter of 8 nm; left panel: STEM image, right panel: snapshot of the 3D tomographic reconstruction.

In order to further examine the NW diameters along their length, electron tomography experiments on two specific NWs were carried out. Figure 4 shows the tomographic reconstruction of hollow WC NWs grown at (a) 7 pA and (b) 2 pA. One can see from the images that the cavities are present up to the tip of the NW. On the left panel of Figure 4a, a STEM image of the NW with outer and inner diameter of 142 and 28 nm, respectively, is shown. On the right panel, a snapshot of the colored 3D tomographic reconstruction is depicted. Figure 4b shows a STEM image of the NW with outer and inner diameter of 77 and 8 nm, respectively, on the left panel, and a snapshot of the colored 3D tomographic reconstruction is displayed on the right panel. Three movies of the tomographic reconstruction for each hollow NW are added in Supporting Information File 1–7, including a transversal (*x*–*y*) and a longitudinal (*y*–*z*) section, and a colored three-dimensional reconstruction.

#### Microstructure

**Figure 5:**
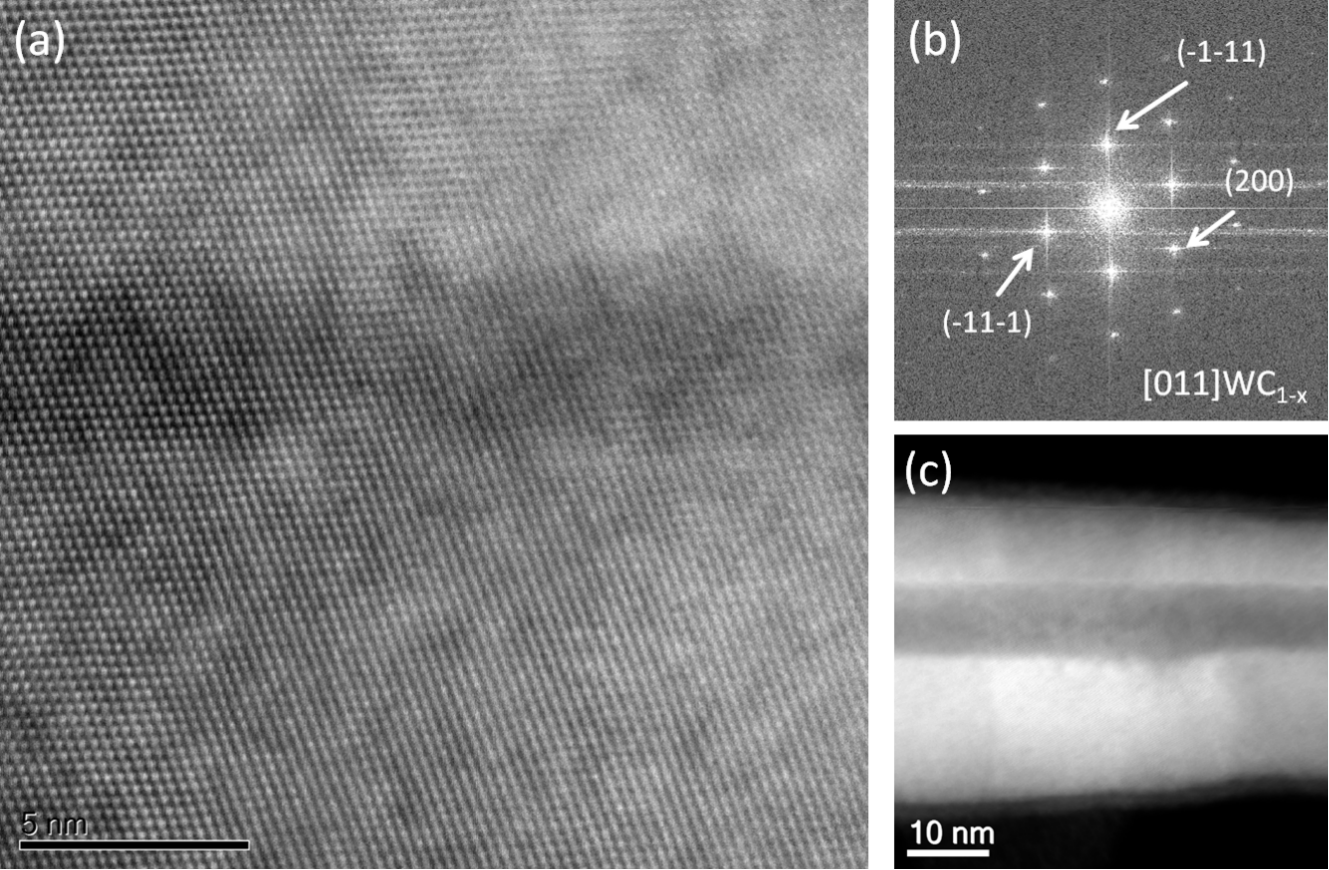
(a) HRSTEM image of a typical hollow WC NW grown at 1.3 pA. (b) Fast Fourier transform of the image in (a), showing the crystalline nature of the material and indexed as the [011] zone axis of the fcc WC_1−_*_x_* structure. (c) Lower magnification STEM image of the WC NW in (a).

Concerning the microstructure of the NWs, high-resolution scanning transmission electron microscopy (HRSTEM) images have been acquired sequentially and processed to extract the crystallographic structure (Figure 5). We indexed the spots indicated in the fast Fourier transform (Figure 5b) of the image in Figure 5a with the planes (−11−1), (−200) and (−1−11), and the [011] zone axis of the WC_1−_*_x_* fcc structure, with a lattice parameter of *a* = 0.4272 nm. A lower magnification STEM image of the NW grown at 1.3 pA is depicted in Figure 5c. These results are in good agreement with the previous work reported by some of the authors [17].

#### Magneto-electrical-transport study

**Figure 6:**
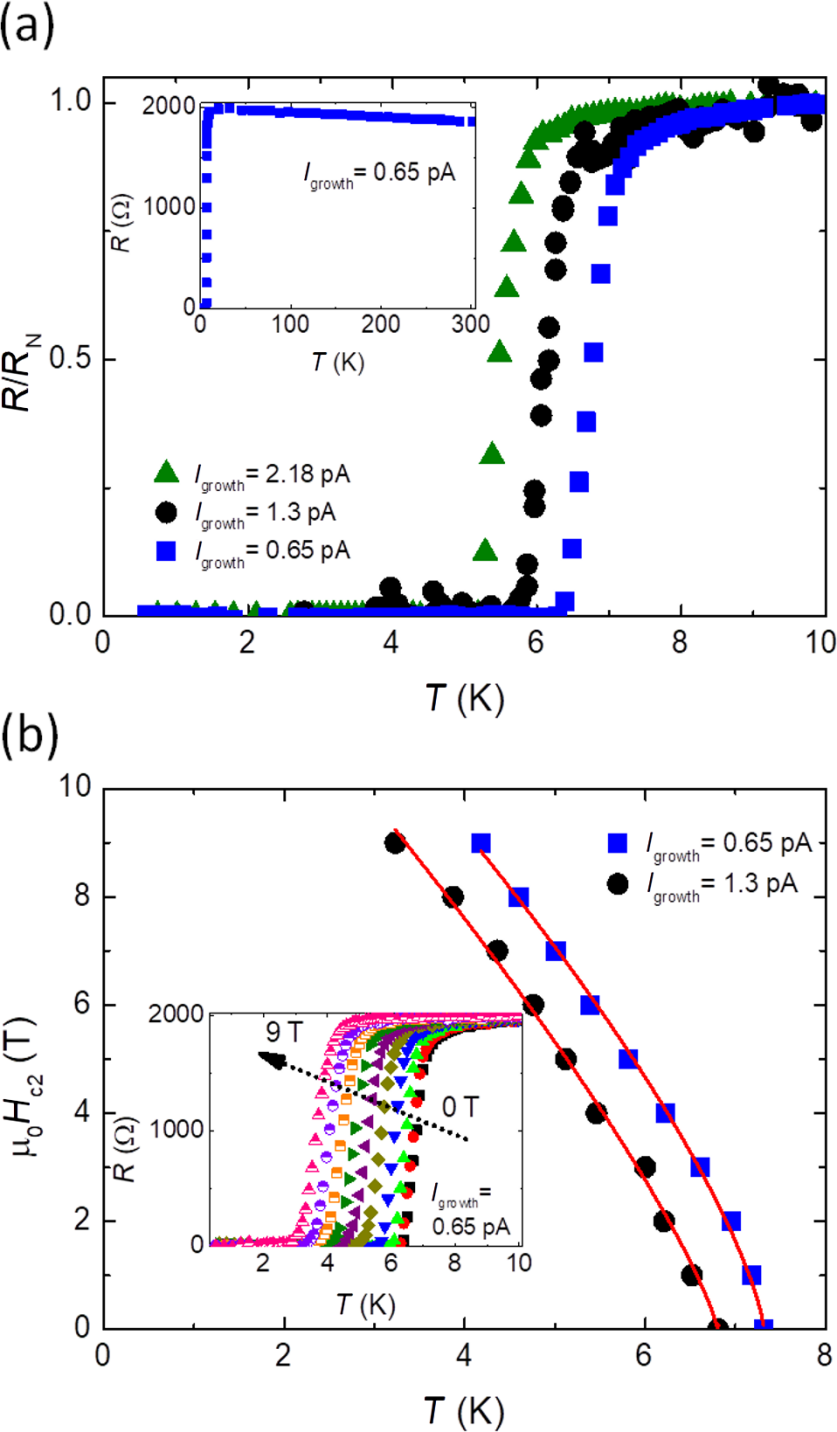
(a) Normalized resistance as a function of the temperature of hollow NWs grown using ion beam currents indicated in the legend. *R*_N_ is the resistance for the normal state at *T* = 10 K. The inset shows the resistance as function of the temperature (full range) for a NW grown at 0.65 pA, *I*_bias_ = 100 nA. (b) Upper critical magnetic field (µ_0_*H*_c2_) as a function of the temperature of NWs grown using ion beam currents indicated in the legend. Data is fitted to a power-law equation. The inset shows the resistance as a function of the temperature for a NW grown at 0.65 pA under perpendicular magnetic fields from 0 to 9 T.

To determine the critical superconducting parameters in NWs grown at 0.65, 1.3, and 2.18 pA (Figure 6 and Table 2), a magneto-electrical transport study using the typical four-point-probe configuration has been performed. Following the procedure described in [17], first 3D NWs were placed flat on the SiO_2_ layer of a Si/SiO_2_ substrate by means of a nano-manipulator. Then, four Pt FIBID contacts were grown to connect the NWs to pre-patterned Ti pads. Finally, we made four-point-probe electrical measurements at low temperature (down to 0.5 K) and under a magnetic field perpendicular to the substrate plane (up to 9 T).

**Table 2:** Superconducting parameters of NWs estimated from experimental magneto-transport measurements.

hollow nanowire type	1	2	3

ion beam current (pA)	0.65	1.30	2.18
outer diameter (nm)	36	41	72
*R*_N_ (Ω)	1959	2101	430
*T*_c_ (K)	6.78	6.16	5.45
µ_0_*H*_c2_ (0 K) (*T*)	14.66	14.48	—
*J*_c_ (0 T) (MA/cm^2^)	0.083 (0.6 K)	0.151 (0.6 K)	0.026 (1 K)
ξ (0 K) (nm)	4.74	4.77	—
λ (0 K) (nm)	839	720	874

The NWs change from the normal to the superconducting state at *T*_c_ (0.5*R*_N_) values between 5.45 and 6.78 K (Figure 6a and Table 2). No clear trend was visible in *T*_c_ values for NWs grown using different currents, although the identified *T*_c_ range is in good agreement with the previously reported results [17]. Also, it is up to 1.5 times higher than that of Ga^+^ FIBID nanostructures of similar dimensions [9]. The inset of Figure 6a shows the measured resistance as a function of the temperature in the full temperature range investigated for a NW grown at 0.65 pA. The value of µ_0_*H*_c2_ as a function of the temperature for NWs grown at 0.65 and 1.3 pA is depicted in Figure 6b. The values of µ_0_*H*_c2_ (0.9*R*_N_) are extracted from the resistance-vs-temperature curves under perpendicular magnetic field (inset of Figure 6b). By fitting µ_0_*H*_c2_(*T*) to a power-law equation µ_0_*H_c_*_2_(*T*) ∝ (1 –*T*/*T*_c_)*^n^*, µ_0_*H*_c2_(0 K) is estimated to be approx. 14.5 T for the different NWs. The coherence length, ξ, at 0 K is around 4.77 nm and the estimated magnetic field penetration depth, λ, [38–39] ranges from 720 to 874 nm. Additionally, *J*_c_ (0.6 K, 0 T) is approx. 0.15 MA/cm^2^.

Summarizing, the estimated superconducting parameters (*T*_c_, µ_0_*H*_c2_, *J*_c_, ξ, λ) for these NWs (Table 2) are mostly compatible with those reported for He^+^ FIBID out-of-plane WC nanotubes [17], nanohelices [19], and in-plane NWs used in hybrid microwave resonators [40]. They are potential building blocks for highly packed 3D nano-resonators, superconducting logic gates [41], quantum switches [42], and single-photon detectors [43–45].

## Conclusion

We report a direct writing methodology to create 3D superconducting hollow NWs with tailored diameters using W(CO)_6_ precursor with a highly focused He^+^ beam. The resulting 3D hollow NWs have inner and outer diameters from 5 to 28 nm and from 36 to 142 nm, respectively, and aspect ratios above 196, which is unachievable by other additive manufacturing methods. The electron tomography study proved that the center hole is present along the whole length of the NWs.

As expected, the microstructure corresponds to the fcc WC_1−_*_x_* phase. By studying their magnetotransport properties, we found *T*_c_ ≈ 6.8 K, as well as µ_0_*H*_c2_ ≈ 14.7 T and *J*_c_ ≈ 0.15 MA/cm^2^. The presented methodology yields an advanced bottom-up approach for the fabrication of innovative 3D nano-architectures, in which nano-superconductivity may provide an advantage, for future electronic components, particularly for sensors, energy-storage components, and quantum computing.

## Experimental

### Growth of 3D hollow WC nanowires

He^+^ FIBID hollow WC NWs have been fabricated in a ZEISS ORION NanoFab instrument equipped with a helium ion beam column and a single-needle gas injection system (GIS) through which W(CO)_6_ gas is delivered to the process chamber.

The NWs were deposited on top of the pre-patterned Ti pads (150 nm in thickness) to prevent charge effects on the insulator layer (250 nm thick of SiO_2_) thermally grown on a silicon wafer [23]. These chips were fabricated following a routine recipe for UV optical lithography using a lift-off method. For the electron tomography and (HR)STEM experiments, NWs were directly grown on Cu TEM grids. Typical deposition conditions used for the He^+^ FIBID process were as follows; precursor material: tungsten hexacarbonyl, W(CO)_6_; *T*_precursor_ = 55 °C; GIS_needle diameter_ ≈ 500 µm; GIS*_z_* ≈ 500 µm; GIS*_x,y_* ≈ 60 µm; *P*_base_ ≈ 3 × 10^−7^ mbar; *P*_process_ ≈ 4 × 10^−6^ mbar; acceleration voltage = 30 kV; pattern shape: spot mode; ion beam current range of 0.54 to 6.47 pA and dose range of 0.1 to 1.4 nC.

### Microstructure and tomography at the nanometer-scale

Scanning transmission electron microscopy (STEM) imaging and EDS were carried out in a probe-corrected FEI Titan 60–300 operated at 300 kV and equipped with a high-brightness X-FEG and a CETCOR *C*_s_ corrector for the condenser system to provide sub-angstrom probe size.

STEM high-angle annular dark-field (HAADF) tomography was performed using a Thermo Fisher Tecnai field-emission gun operated at 300 kV. The angular range for the tilt series was ±70° with pictures taken every 1°. Image alignment and 3D reconstruction was carried out with FEI tomography acquisition software Inspect 3D after the acquisition of 140 images. The movies of the tomographic reconstruction for each hollow NW were performed using Amira 3D software.

### Magneto-electrical transport study

The magneto-electrical transport measurements on the NWs were carried out using a ''Physical Property Measurement System'' (PPMS), from Quantum Design equipped with a helium-3 refrigerator insert.

## Supporting Information

Movies of 3D tomographic reconstruction.

File 1 Electron tomography_3D reconstruction_hollow NW grown at 2 pA and 0.6 nC.

File 2 Electron tomography_3D longitudinal_hollow NW grown at 2 pA and 0.6 nC.

File 3 Electron tomography_longitudinal section_hollow NW grown at 2 pA and 0.6 nC.

File 4 Electron tomography_transversal section_hollow NW grown at 2 pA and 0.6 nC.

File 5 Electron tomography_3D reconstruction_hollow NW grown at 7 pA and 1.009 nC.

File 6 Electron tomography_3D longitudinal_hollow NW grown at 7 pA and 1.009 nC.

File 7 Electron tomography_transversal section_hollow NW grown at 7 pA and 1.009 nC.
